# Rare Appendicitis-Like Syndrome: The Case of the Obstructing Broccoli

**DOI:** 10.1155/2014/764869

**Published:** 2014-04-22

**Authors:** Stephen Jones, Patrick Narh-Martey, Mitun Patel, Amanpreet Dhaliwal, Jessica Persson, Denis Orr

**Affiliations:** Department of Surgery, Western Reserve Health Education, USA

## Abstract

The diagnosis of acute appendicitis can be somewhat obscure in a patient that presents with right lower quadrant abdominal pain. The advancement and ease of imaging have made CT scanning readily available in the emergency department. Management can be challenging when the patient has a high likelihood of appendicitis based on clinical suspicion and negative CT scan. The purpose of this case report is to demonstrate how an obstructing bezoar caused an appendicitis-like syndrome in a patient with negative CT scan and clinical diagnosis of acute appendicitis. This case report will discuss the appendicitis-like syndrome of an obstructing bezoar and an approach at management.

## 1. Introduction

The diagnosis of acute appendicitis can be somewhat obscure in a patient that presents with right lower quadrant abdominal pain. Pyrexia, pain localized to McBurney's point, and nausea can be a common occurrence in the emergency department. A good history and physical exam often make the diagnosis and negate the need for radiologic imaging. However, the advancement and ease of imaging have made CT scanning readily available in the emergency department. The sensitivity of CT scans for identifying acute appendicitis reaches approximately 97% and the negative predictive value (NPV) reaches 99% by some reports [1, 2]. Management can be challenging when the patient has a high likelihood of appendicitis based on clinical suspicion and negative CT scan. The purpose of this case report is to demonstrate how obstructing bezoars can cause an appendicitis-like syndrome in a patient with negative CT scan and clinical diagnosis of acute appendicitis. Foreign body ingestion is common and typically passed without intervention [3]. Depending on the type of foreign body, presentation can vary. Impaction, bowel obstruction, and perforation are the most common presentations described [3]. This report will discuss the appendicitis-like syndrome of an obstructing bezoar and an approach at management.

## 2. Case Report

### 2.1. Case Presentation

A 32-year-old otherwise healthy male presents to the emergency department with a history of acute onset right lower quadrant abdominal pain which is 10 hours in duration. He describes the pain as throbbing in nature, 7 out of 10 on the pain scale with no radiation. No alleviating or exacerbating factors were described. The patient also complains of nausea and vomiting for this time period as well. In addition, he reports subjective fever and chills. He has no sick contacts. The patient states that his last meal was the past night and he reports having broccoli for dinner. He reports no difficulty in chewing food. He denies any significant past medical or surgical history including any gastric surgery or gastrointestinal resection. There is no history of Crohn's disease or ulcerative colitis in his family.

### 2.2. Physical Exam

*General Appearance.* He is alert and oriented times 3, in mild distress.

*Vital Signs.* Temperature was 98.2 F. Blood pressure was 112/65 mmHg. Pulse was 96 bpm. Respirations were 16. SPO2 was 99% on room air.

*Skin.* Skin was warm and dry. HEENT indicated the following: normocephalic/atraumatic, PERRL, moist oral mucosa, and healthy dentition. Lungs were clear to auscultation bilaterally with no wheezes, rales, or rhonchi. Heart had regular rate and rhythm and no murmurs, rubs, or gallops.

*Abdomen.* Abdomen was soft. He is not distended. He is tender to palpation in the right lower quadrant with positive rebound at McBurney's point, positive Rovsing sign, and positive Obturator sign. No hernias were identified. Rectal exam revealed good anal sphincter tone, no external hemorrhoids/skin tags, and hemoccult negative stool.

### 2.3. Labs

See Figure 1.

### 2.4. CT Findings

See Figures 2(a) and 2(b).

### 2.5. The Operation

Needle was inserted at the umbilicus and pneumoinsufflation was obtained. Using OptiView guidance, the laparoscope was inserted through a trocar into the abdominal cavity. Next, the area was inspected for injury, failing to reveal any. Additional ports were placed. The diagnostic laparoscopy immediately revealed exudative fluid in the abdominal cavity. This was yellow-green. It was suctioned and the specimen was sent for Gram stain culture and sensitivity. The fluid was washed out. The appendix was identified and found to be relatively normal with minimal serositis at the tip. The small bowel had serositis and inflammation throughout the majority of the small bowel affecting the proximal bowel more than the distal bowel. The terminal ileum was then identified from the ileocecal valve proximally and identification of a mass in the small bowel was made. The mass was firm and impacted and not able to be significantly milked in either direction. The stomach was inspected along with the duodenum and the gallbladder. The gallbladder was distended, but no signs of acute cholecystitis were appreciated. Next, anesthesia administered air to the stomach as well as methylene blue and neither of these tests revealed a leak in the stomach or duodenum. Therefore, the small bowel was then addressed just distally to the impaction and enterotomy was made. This was done by scoring the small bowel laparoscopically. The decision was made to open the fascia slightly at the umbilical port site and withdraw the small bowel. In doing so, the impacted foreign body had been milked away from the site. The bowel holding the foreign body was unable to be extracted through the enlarged port site, and, therefore, towel clamps were applied. Pneumoinsufflation was reobtained. The bowel was grasped with the grasper and extracted at the umbilical port site. The small bowel was opened with monopolar cautery via the Bovie cautery extracorporeally and then dropped back into the abdominal cavity. Towel clamps were applied to the skin and the impacted foreign body was milked towards the enterotomy (Figure 4). This technique revealed a large unchewed and undigested piece of broccoli measuring approximately 5-6 centimeters in diameter at widest point (Figure 5). The food piece was then scooped up with a bag through the midline port site, which was held in place with the towel clamps and extracted extracorporeally. The bowel was then extracted extracorporeally and the bowel was closed using running 0 Vicryl. After testing the integrity of the enterotomy site, additional Lembert interrupted vertical sutures were placed to reinforce the area. Next, the abdominal cavity was washed out with approximately 1.5 liters of warm saline. The fascia was closed using running 0 PDS sutures, and the skin was closed with subcuticular stitches. The patient tolerated the procedure well (Figure 3).

## 3. Discussion

Foreign body ingestion is a common problem experienced in the emergency department. The ingestion of foreign objects is most commonly seen in children, while, on the other hand, adult presentations of foreign body ingestion are more likely to be due to bezoars. A bezoar is defined as a swallowed mass of undigested material found within the gastrointestinal tract [4]. The most typical compositions of bezoars include hair (as seen in trichophagia), vegetable fibers, or, interestingly, persimmon fruit. Bezoars are uncommon and estimated to occur in about 0.4% of the general population [5]. At least 80% of ingested foreign objects are passed spontaneously through the gastrointestinal tract without complication [3]. Although rare, bezoars are a recognized cause of intraluminal bowel obstruction [6]. Other complications associated with bezoars and foreign body ingestion include bleeding, impaction, obstruction, and perforation [3]. These complications tend to occur at areas of angulation or narrowing of the gastrointestinal tract [7]. Therefore, patients at increased risk of bezoar formation include those with reduced gastrointestinal motility or altered anatomy status after gastric surgery, most notably for treatment of peptic ulcer disease [4, 8].

Accurate preoperative diagnosis of bezoars as the cause of a patient's acute abdomen symptoms is difficult [9]. Due to low incidence, bezoars are naturally on the bottom of the list for differential diagnoses [4]. Various methods of imaging can be utilized to aid in making a diagnosis. Radiologic options such as abdominal X-ray, barium studies, and abdominal ultrasound may be useful to diagnose intestinal obstruction, but they are not helpful in identifying a specific cause for obstruction [4]. The imaging tool of choice for diagnosing bezoars in the preoperative period is CT examination [4]. The treatment of choice for bezoars is surgical intervention, specifically laparoscopic surgery [10]. Imaging is a valuable tool for diagnosing acute appendicitis or intestinal obstruction and revealing a different etiology of a patient's clinical presentation. However, even with CT imaging, bezoars can be radiolucent or unidentifiable, as in this case report. Clinical judgment is then required to determine the next step in management.

Although clinical presentations of bezoars are unusual, there have been reports of foreign body ingestion mimicking other diseases such as renal stones and irritable bowel syndrome [11]. Furthermore, there are also few reports of bezoars obstructing the appendiceal lumen causing appendicitis [12].

However, to our knowledge, this is one of the first case reports of acute appendicitis-like syndrome caused by an intestinal obstructing bezoar. While bezoars are rare, they need to be part of a working diagnosis when dealing with atypical presentations, especially negative CT imaging, of intestinal obstruction and acute appendicitis.

## 4. Conclusion

Foreign body ingestion in children and acute appendicitis are both common problems experienced in the emergency department, while bezoars in adults are uncommon and estimated to occur in about 0.4% of the general population. Complications of bezoars include intestinal obstruction and, in this case report, an acute appendicitis-like syndrome. Accurate preoperative diagnosis of bezoars as the cause of a patient's acute abdomen is difficult. CT examination is a valuable tool for diagnosing acute appendicitis or intestinal obstruction and revealing the etiology of a patient's clinical presentation. However, even with CT imaging, bezoars can be radiolucent or unidentifiable, as seen in this case report. Clinical judgment is therefore required to determine the next step in management. This case report describes the rare clinical presentation of an obstructing bezoar causing an acute appendicitis-like syndrome.

## Figures and Tables

**Figure 1:**
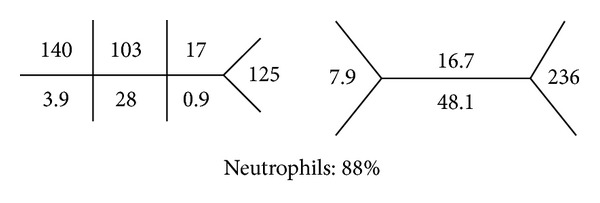


**Figure 2:**
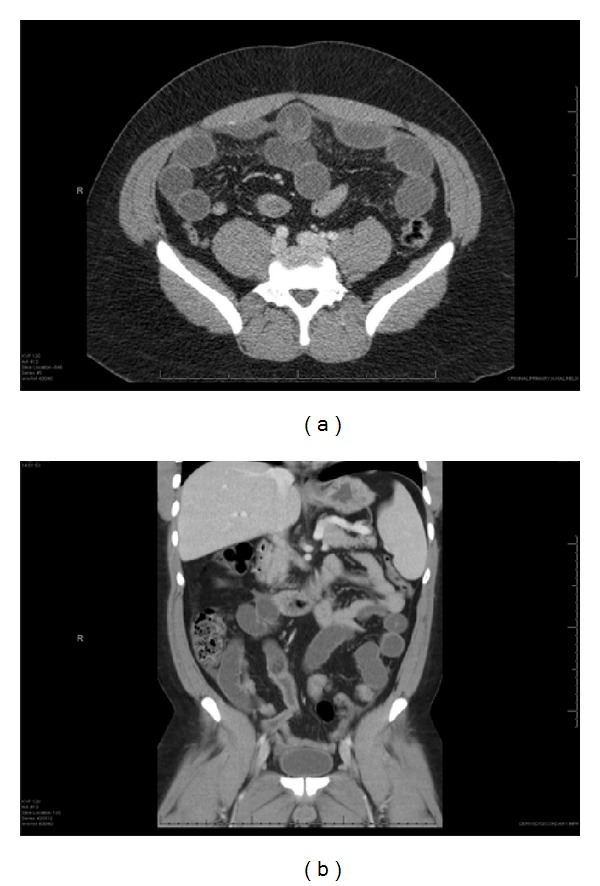


**Figure 3:**
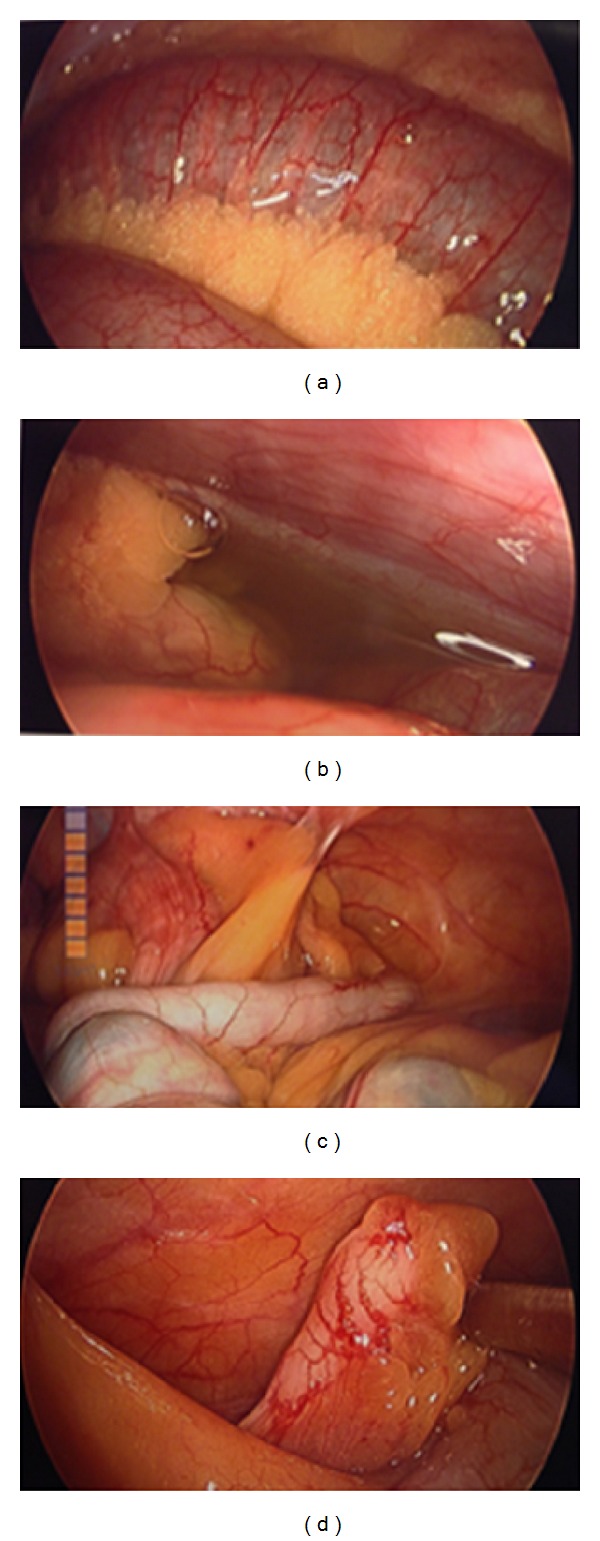
(a) Mildly dilatated small bowel, (b) turbid fluid paracolic gutter, (c) normal appendix, and (d) serositis at tip of appendix.

**Figure 4:**
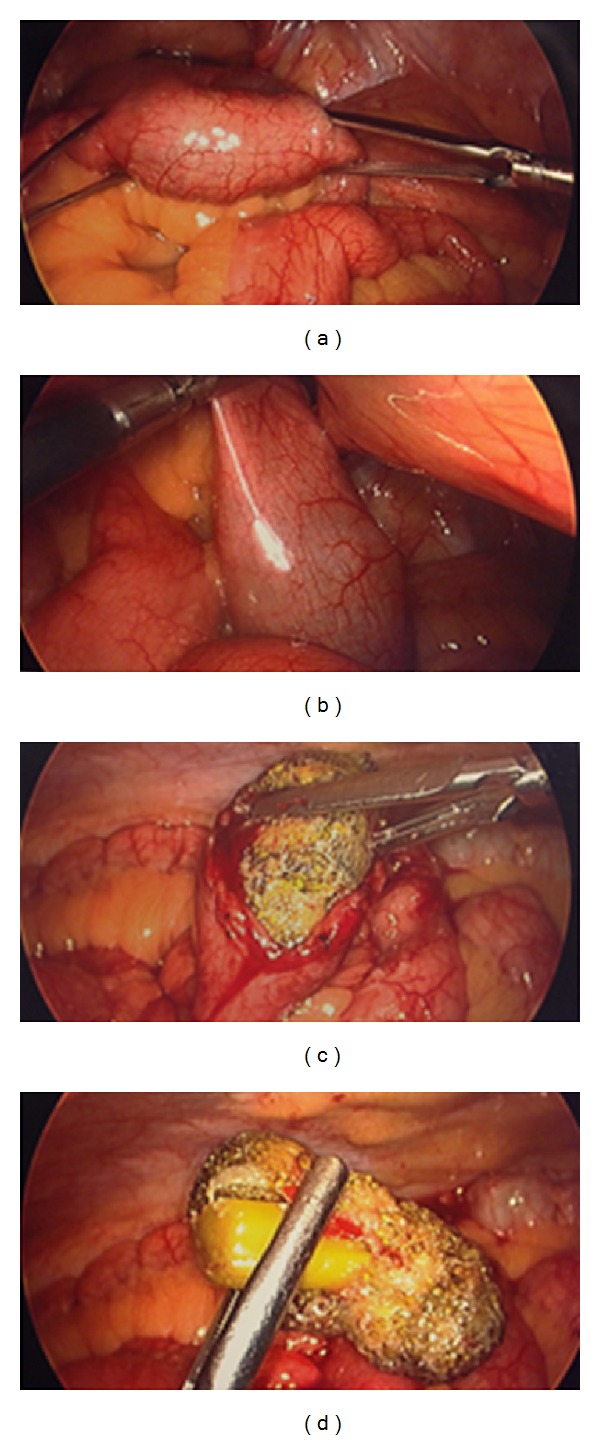
(a) Intraluminal mass distal small bowel, (b) intraluminal mass distal small bowel, (c) extraction of broccoli from small bowel, and (d) undigested/unchewed piece of broccoli.

**Figure 5:**
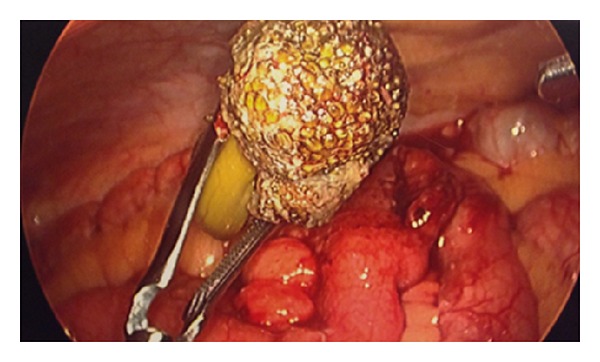
Large 5 cm × 6 cm piece of broccoli.
